# Acute emergency care and airway management of caustic ingestion in adults: single center observational study

**DOI:** 10.1186/s13049-016-0240-5

**Published:** 2016-04-11

**Authors:** Manuel F. Struck, André Beilicke, Albrecht Hoffmeister, Ines Gockel, André Gries, Hermann Wrigge, Michael Bernhard

**Affiliations:** Department of Anaesthesiology and Intensive Care Medicine, University Hospital Leipzig, Liebigstr. 20, 04103 Leipzig, Germany; Department of Gastroenterology and Rheumatology, Section of Interdisciplinary Endoscopy and Sonography, University Hospital Leipzig, Leipzig, Germany; Department of Visceral, Transplant, Thoracic and Vascular Surgery, University Hospital Leipzig, Leipzig, Germany; Emergency Department, University Hospital Leipzig, Leipzig, Germany

**Keywords:** Caustic ingestion, Intoxication, Emergency management, Airway management, Endoscopic management

## Abstract

**Background:**

Caustic ingestions are rare but potentially life-threatening events requiring multidisciplinary emergency approaches. Although particularly respiratory functions may be impaired after caustic ingestions, studies involving acute emergency care are scarce. The goal of this study was to explore acute emergency care with respect to airway management and emergency department (ED) infrastructures.

**Methods:**

We retrospectively evaluated adult patients after caustic ingestions admitted to our university hospital over a 10-year period (2005–2014). Prognostic analysis included age, morbidity, ingested agent, airway management, interventions (endoscopy findings, computed tomography (CT), surgical procedures), intensive care unit (ICU) admission, length of stay in hospital and hospital mortality.

**Results:**

Twenty-eight patients with caustic ingestions were included in the analysis of which 18 (64 %) had suicidal intentions. Ingested agents were caustic alkalis (*n* = 22; 79 %) and acids (*n* = 6; 21 %). ICU admission was required in 20 patients (71 %). Fourteen patients (50 %) underwent tracheal intubation and mechanical ventilation, of which 3 (21 %) presented with difficult airways. Seven patients (25 %) underwent tracheotomy including one requiring awake tracheotomy due to progressive upper airway obstruction. Esophagogastroduodenoscopy (EGD) was performed in 21 patients (75 %) and 11 (39 %) underwent CT examination. Five patients (18 %) required emergency surgery with a mortality of 60 %. Overall hospital mortality was 18 % whereas the need for tracheal intubation (*P* = 0.012), CT-diagnostic (*P* = 0.001), higher EGD score (*P* = 0.006), tracheotomy (*P* = 0.048), and surgical interventions (*P* = 0.005) were significantly associated with mortality.

**Conclusions:**

Caustic ingestions in adult patients require an ED infrastructure providing 24/7-availability of expertise in establishing emergent airway safety, endoscopic examination (EGD and bronchoscopy), and CT diagnostic, intensive care and emergency esophageal surgery. We recommend that - even in patients with apparently stable clinical conditions - careful monitoring of respiratory functions should be considered as long as diagnostic work-up is completed.

## Background

Ingestion of caustic substances is a rare emergency that may cause severe injuries to the upper digestive tract and to the laryngopharynx. In contrast to pediatric populations, caustic ingestions in adults are less frequent, but more often life-threatening and associated with psychiatric comorbidity [1–3]. The characteristics and pathophysiology of tissue damage after caustic ingestion depends on its nature (alkali or acid), concentration, contact time and volume of the agent, whereas the likelihood of perforation of the esophageal, gastric and intestinal wall cannot be reliably predicted. Involvement of the upper airway and subsequent respiratory failure due to laryngopharyngeal edema or tracheal aspiration belong to the most dangerous complications in the acute phase [1–6].

After cardiopulmonary stabilization, early esophagogastroduodenoscopy (EGD) and/or computed tomography (CT), and intensive care unit (ICU) monitoring are widely recommended, whereas gastric lavage, emesis induction, charcoal use, neutralization agents and deliberate use of steroids and antibiotics should be avoided [1–4]. Clear evidence about the role of proton pump inhibitors and histamine blockers is still lacking although frequently performed. Currently, emergency management of caustic ingestions depends on local treatment protocols rather than on specific guidelines. Randomized controlled trials upon emergency management of caustic ingestions are not available and published literature mainly focuses on animal experiments, diagnostic methodology and surgical outcomes [1–3]. The goal of this study was to explore acute emergency care particularly with respect to airway management and emergency department (ED) infrastructures.

## Methods

After approval of the ethics committee (No. 137-15-20042015), the database of the University Hospital Leipzig was reviewed in order to identify patients who were classified by the ICD-10 system for caustic injuries and chemical burns (ICD-10 code T27.x and T28.x) between 01/2005 and 12/2014. Caustic ingestion was defined as oral swallowing of corrosive substances either accidentally or by suicidal intention. Patients who sustained just oropharyngeal contact without incorporation, with incomplete documentation, and being under 18 years of age were excluded. The University Hospital Leipzig is a 1350-beds academic medical center providing advanced emergency care for approximately 1 million people including the inhabitants of the City of Leipzig and the region of West Saxony, Germany. All interdisciplinary specialists for the treatment of caustic ingestions are available 24/7, i.e. emergency physicians, anesthetists, endoscopy teams (gastroenterologists and pulmologists), radiologists, otolaryngologists, psychiatrists, intensivists and upper gastrointenstinal (GI) surgeons. First-line equipment for ED airway management in our center includes Macintosh blades, stylets and tube exchangers, laryngeal masks, laryngeal tubes, and cricothyrotomy sets. On demand, video laryngoscopes, and bronchoscopes for fiberoptic intubation purposes are available in various sizes.

### General management

Patients were admitted to the ED by emergency medical services (EMS) from the scene of the accident or by interhospital transfer from smaller hospitals. The ED provided an interdisciplinary medical team for patient triage, diagnosis and emergency treatment. Depending on clinical examination, laboratory results and endoscopy and/or CT results, patients were scheduled for either medical or surgical ICU. Patients with less severe symptoms could be transferred to normal wards under discretion of the attending emergency physician after interdisciplinary consultation.

We analyzed demographic, diagnostic and treatment specific parameters, identified potential individual risk factors (e.g. age, caustic agent, volume of caustic agent, psychiatric co-morbidity, intensive care and surgery) and obtained variables characterizing emergency care, intensive care, complications, and hospital mortality. Data were obtained from the paper-based or electronic charts.

### Statistical analysis

Data are reported as median (min, max, or interquartile range), mean ± standard deviation, and counts (percentage). Statistical comparisons between survivors and non-survivors were performed using the χ^2^ test for qualitative, and Student’s *t* test or Mann-Whitney U-test for quantitative data. The alpha level of significance was set at 0.05. All tests were two-tailed. Univariate analysis was performed to identify independent predictors of tracheal intubation and mortality. Variables tested included age, gender, ingested substance (alkalis vs. acids), intention (suicidal vs. accidental), psychiatric history, history of alcohol abuse, tracheal intubation, tracheotomy, mechanical ventilation (days), bronchoscopy, otolaryngology examination, endoscopy stage, computed tomography (CT), intensive care unit (ICU) admission, and surgical interventions. Multivariate analysis was not performed because of small sample size.

## Results

During the study period, 103 patients were classified to ICD-10 code T27x and T28x. According to a detailed case-by-case chart review (electronic and/or paper-based) 19 patients had only oral contact with corrosive substances, visited the hospital with the same ICD-10 code repetitively (21 patients), were aged <18 years (23 patients), and/or were encoded incorrectly (12 patients). Twenty-eight patients (27 %) met the inclusion criteria and had complete datasets of new and confirmed caustic ingestion and thus were subject of the study. Demographic data of patients are provided in Table 1.

**Table 1:** Demographic data of patients after caustic ingestion

	Total (*n* = 28)	Survivors (*n* = 23)	Non-survivors (*n* = 5)	*P*
Age, years; median (interquartile range)	44,5 (31;56)	45 (31;54)	44 (43;85)	0.294
Male; n (%)	16 (57)	14 (61)	2 (40)	0.412
Ingested agents; n (%)				0.935
alkalis	22 (79)	18 (78)	4 (80)	
acids	6 (21)	5 (22)	1 (20)	
Intention; n (%)				0.437
suicidal	18 (64)	14 (60)	4 (80)	
accidental	10 (35)	9 (39)	1 (20)	
Psychiatric diagnosis; n (%)				0.092
Yes, schizophrenia	3 (10)	1 (4)	2 (40)	
Yes, depression	6 (21)	5 (21)	1 (20)	
No, affect, appellative	10 (35)	9 (39)	1 (20)	
No, mental healthy	9 (32)	8 (34)	1 (20)	
Previous suicide attempts; n (%)	6 (21)	3 (13)	3 (60)	0.437
History of alcoholism; n (%)	7 (25)	6 (26)	1 (20)	0.266
Tracheal intubation; n (%)	14 (50)	9 (39)	5 (100)	0.012
Tracheotomy; n (%)	7 (25)	4^a^ (17)	3 (60)	0.048
Mechanical ventilation, days; mean (SD)	9.93 (10.48)	6.5 (9.14)	16 (10.89)	0.108
Bronchoscopy; n (%)	12 (40)	11 (47.8)	4 (80)	0.068
Otolaryngology examination; n (%)	13 (46)	10 (43)	3 (60)	0.520
EGD, n (%)	21 (75)	17 (61)	4 (80)	0.786
Zargar score				0.006
grade I; n (%)	2 (10)	2 (12)	0 (0)	
grade IIa; n (%)	5 (24)	5 (29)	0 (0)	
grade IIb; n (%)	8 (38)	7 (41)	1 (25)	
grade IIIa; n (%)	2 (10)	2 (12)	0 (0)	
grade IIIb; n (%)	4 (19)	1 (6)	3 (75)	
CT diagnostic; n (%)	11 (39)	6 (26)	5 (100)	0.001
Emergency surgery	5 (17)	2 (8)	3 (60)	0.005
LOS ICU, days; mean (SD)	8.57 (12.12)	8.60 (9.93)	22.20 (19.36)	0.128
LOS hospital, days; mean (SD)	17.35 (20.31)	15.08 (19.97)	27.60 (20.60)	0.123

*SD* standard deviation, ^a^one patient undergoing awake tracheotomy in the emergency department, *EGD* Esophagogastroduodenoscopy (Zargar classification [7]), *CT* computed tomography, *LOS* length of stay, *ICU* intensive care unit

### Patient’s characteristics

There were 16 male patients (57 %) and median age was 44.5 years (range 20–91 years). Seven patients were admitted from smaller hospitals (all but 2 patients at the day of ingestion). Suicidal intention was accounted for 18 patients (64 %), of whom 9 (50 %) had existing psychiatric history (depression, *n* = 6, and schizophrenia, *n* = 3). Six patients had known suicide attempts in their medical history (1 in the non-suicide group), 4 patients presented with previous typical non-suicidal self-injury (forearm incisions) and 1 patient presented with accompanying severe suicidal stabbing injuries to the chest. A history of alcoholism was present in 6 patients (21 %). Ingested substances were caustic alkalis (*n* = 22; 79 %) and acids (*n* = 6; 21 %). Alkali substances were strong alkalis (oven cleaner, natrium hydroxide and calcium hydroxide) in 13 patients and weak alkalis (household cleaners and natrium bicarbonate) in 8 patients. Ingested acids were strong acids (hydrochloric acid and battery acid) in 3 patients and weak acids (citric acid and vinegar) in 3 patients. In 21 patients (75 %), median ingested volume was 200 ml (range 20–700 ml), and for 7 patients (25 %) ingested volumes remained unknown. Moreover, 3 patients combined caustic agents with other toxic substances for ingestion (liquid fertilizer, laundry detergent and *Convallaria majalis*, respectively).

### Airway management

Tracheal intubation was required in 14 patients (50 %). In 1 patient, tracheal intubation had been performed by prehospital physician-staffed EMS. Three patients had already been intubated by the transferring hospitals. In 6 patients intubation was performed in the ED, and 1 patient required awake tracheotomy as primary airway management due to severe laryngeal injury and progressive upper airway obstruction. One patient was primarily scheduled to the normal ward, suffered severe respiratory failure and underwent delayed intubation by the in-house emergency medical team, resulting in hypoxemic brain damage. Another 3 patients required tracheal intubation after ICU admission. From all 14 patients who underwent tracheal intubation, 3 (21 %) had documented difficult airways. All of them presented grade III according to the Cormack/Lehane classification, but were successfully intubated within two attempts using Macintosh blades. The need for tracheal intubation was significantly associated with mortality (*P* = 0.012).

### Diagnostic procedures

Emergency EGD was performed in 21 patients (75 %). Patients were staged according to the Zargar classification [7]. Zargar lesions grade IIIb were significantly associated with mortality (*P* = 0.006). In 2 patients, EGD could not be completed due to incompliance of the patient. From the 7 patients without EGD, 4 were considered clinically stable, 2 refused endoscopic examinations, and 1 underwent CT and died before EGD. Eleven patients (39 %) underwent CT examinations that appeared to be significantly associated with mortality (*P* = 0.001). In 1 patient, severe caustic ingestion remained undetected until CT that was performed because of multiple stabbing injuries to the chest in a coincidental suicide attempt. Furthermore, CT examinations of 2 patients after caustic ingestion led to unexpected diagnoses of pulmonary artery embolism (*n* = 1) and prostate carcinoma (*n* = 1). One patient presented with pneumomediastinum in a control CT examination 6 weeks after caustic injury.

### Respiratory intensive care treatment

Out of the 28 patients, 20 (71 %) were admitted to the ICU, in 11 cases to the surgical ICU and in 9 to the medical ICU. Of these, 14 intubated patients (70 %) required a mean 9.93 ± 10.48 days of mechanical ventilation of which 10 underwent frequent bronchoscopic examinations (Table 1). In 3 patients, tracheal caustic aspiration was confirmed by bronchoscopy whereas 1 of these developed tracheal rupture and died of severe mediastinitis. Apart from 1 patient who required awake tracheotomy in the ED, tracheotomy was performed in another 6 patients at the ICU after a median of 3 days (range 0–9 days) (*P* = 0.048).

### Surgical interventions

Emergency surgery was performed in 5 patients (18 %) within a median of 6 h (range 4–16 h) after admission (Table 1). Esophagogastrectomy was performed in 4 patients and was extended to other abdominal organs in 3 patients: bowel (*n* = 2), gallbladder (*n* = 2), doudenopancreas (*n* = 1), and colon (*n* = 1). Esophagectomy with gastric preservation was performed in 1 patient. Emergency surgery was significantly associated with mortality (*P* = 0.005). Two patients (40 %) survived the emergency operation and underwent reconstructive surgery. One patient underwent gastric pull-up with cervical anastomosis 2 months after ingestion. The other patient underwent bilateral neck-dissection, restorative jejunopharyngoplasty and cervical radialis flap after 8 months and 13 months, respectively.

### Supportive care and outcomes

All but 1 patient (96 %) (who died on the day of admission at the ICU) received high dose intravenous proton pump inhibitor treatment (80 mg/24 h pantoprazole or esomeprazole, respectively). Sepsis occurred in 8 patients (29 %) and antibiotic therapy was performed in 13 patients (46 %) with following substances: cephalosporins (*n* = 4), piperacilline/tazobactam (*n* = 3), meropenem/vancomycin (*n* = 3), imipenem (*n* = 2), ampicilline/sulbactam (*n* = 1), ciprofloxacine (*n* = 1). Enteral nutrition via jejunostomy was performed in 7 patients (25 %). One patient received percutaneous endoscopic gastrostomy. Mean length of stay in the ICU was 8.57 ± 12.12 days and mean length of stay in the hospital was 17.35 ± 20.31 days (Table 1). Four patients (14 %) left the hospital against medical advice and 5 patients (18 %) were transferred to closed psychiatric wards after acute care where 1 of them committed another suicide attempt by drinking a bottle of hand disinfective. Suicidal intention was significantly associated with higher injury severity and mortality (*P* = 0.005). Overall hospital mortality was 18 % whereas 3 patients (60 %) died from sepsis and 2 (40 %) from acute cardiopulmonary decompensation.

## Discussion

The results of our study suggest that caustic ingestions in adults may present with considerable varieties of morbidity and likewise high proportions of less severe conditions (13 patients were discharged within 7 days including 6 patients within 4 days). However, 20 patients were admitted to the ICU, of whom 14 required tracheal intubation. Although only 1 patient underwent prehospital rapid sequence intubation, the development of upper airway edema may rapidly progress after caustic contact to oropharyngeal tissue. Challenging conditions to be expected include erosive ulceration, sloughing and bleeding of oral mucosa, tongue and epiglottis, laryngopharyngeal edema, hypersalivation, subcutaneous emphysema, stridor and respiratory distress [1–6]. This may be reflected in 21 % of our intubated patients who presented with difficult laryngoscopy conditions. Emesis after caustic ingestion should be avoided because it may lead to esophageal and oropharyngeal re-exposition of the caustic substance, tissue perforation due to mechanical forces, and tracheal aspiration of caustic substances. Thus, preventive measures (upright positioning of the patient, antiemetic medication) may be useful [1]. As in our patients, 6 required intubation and 1 emergency tracheotomy in the ED, emergency physicians should involve anesthetists for difficult airway management early [8–11]. Although all patients in this study underwent conventional laryngoscopy using Macintosh-blades, videolaryngoscopy or awake fiberoptic intubation may be considered [1, 2, 8, 11]. Moreover, in this special patient collective, otolaryngologists or general surgeons familiar with awake tracheotomy should be available for surgical airway approaches [12, 13]. Severely compromised patients may benefit from controlled primary tracheotomy in local anesthesia (1 of our patients) [1, 14]. In our patients, tracheotomy was performed due to expected long-term intensive care and ventilator dependence, whereas tracheotomy may also be useful in patients requiring repetitive surgery to avoid oropharyngeal injuries due to intubation procedures [3]. Tongue and mucosal structures may present as black eschar, accompanied by white fibrin exudates, depending on the ingested agent. Affected mucosa frequently presents highly vulnerable and any manipulation may provoke bleeding and swelling in these patients. Early otolaryngology diagnostic should be initiated to evaluate laryngo-pharyngeal mucosa and to confirm possible impairment of the swallowing act. We presume that the mechanism of glottis sealing still works after caustic ingestion in most cases. This protective pharyngeal-glottic mechanism may be altered by impaired consciousness (i.e. substance abuse).

**Fig. 1:**
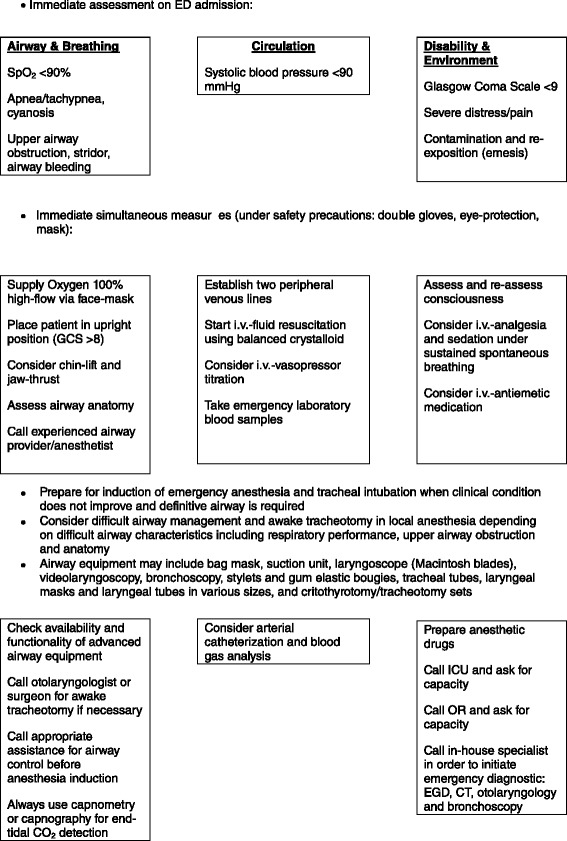
Emergency approach to patients suspect of caustic ingestion with respect to airway management

The relatively high rate of tracheal intubation in this study might be the result of our consequent compliance to national recommendations to initiate emergency anesthesia and airway management to minimize the risk of airway failure [8–11]. The criteria for tracheal intubation in our patients were Glasgow coma scale score <9, pain, bleeding, stridor, and respiratory exhaustion. Due to high varieties of clinical presentation and relatively low sample sizes in our investigation we recommend a careful case-by-case decision making process considering all circumstances of the individual emergency regarding airway management. Nevertheless, it should be kept in mind that tracheal intubation may result in possible difficult airway scenarios, prolonged respirator dependence (and thus risk of ventilator-associated pneumonia) and mechanical destabilisation/stress of already affected pharyngo-laryngeal tissue (risk of bleeding/perforation). An airway management algorithm for patients with caustic ingestion has been provided in Fig. 1.

There may be a risk of misdiagnosis and undertreatment in patients admitted to the ED after caustic injury due to discrepancy of apparent clinical impression and actual disease severity [1, 2, 15]. Patients may appear stable despite considerable illness and thus may deteriorate rapidly and severely in due course (1 of our patients). However, acute damage of the subglottic airway due to caustic aspiration or tracheobronchial rupture (posterior necrosis) has been reported rarely at ED admission whereas microaspiration, ventilator associated pneumonia and late tracheobronchial rupture may occur more frequently as late complications during ICU treatment (Table 2) [16–20]. We observed three cases of tracheal caustic aspiration including one case of tracheobronchial rupture detected by repeated bronchoscopy. Timing of tracheobronchial perforation is not predictable and has been reported as long as 20 days (own data) and even 32 days after ingestions, respectively [16, 17]. In the literature, tracheobronchial perforation, either caused by tracheal caustic aspiration, or by full thickness necrosis of neighbouring esophageal structures, deteriorate prognosis significantly. Benjamin et al. reported 20 patients who underwent emergency treatment of tracheobronchial necrosis after caustic ingestion of whom 9 (45 %) died including 2 with intraoperative fatalities [18]. Another 21 patients have been reported with a mortality rate of 75 % [19]. Due to anatomy, tracheobronchial perforation usually appears along the distal trachea, carinal region and left mainstem bronchus [18].

**Table 2:** Selected case series of airway involvement (>10 patients) due to caustic ingestion in adult patients

Study	Origin	Total patients	Intentional ingestion	Kind of airway involvement	Airway reconstruction	Mortality
Benjamin et al. 2015 [18]	France	*n* = 1280	*n* = 20 (100 %)	Tracheobronchial necrosis early (on admission) *n* = 14, late (postoperatively) *n* = 7	Pulmonary patch repair (*n* = 16)	Airway related: *n* = 9 (45 %)
Sarfati et al. 1992 [19]	France	*n* = 679	unknown	Tracheobronchial necrosis *n* = 21	Pulmonary patch repair (*n* = 6)	Airway related: 75 %
Lurie et al. 2013 [26]	Israel	*n* = 23	*n* = 18 (78 %)	Aspiration *n* = 11	none	Total: *n* = 5 (22 %)
Cheng et al. 2008 [27]	Taiwan	*n* = 273	*n* = 194 (71 %)	Aspiration pneumonia *n* = 31	none	Total: *n* = 17 (7 %)
				Respiratory failure *n* = 21		

Immediate measure to manage airway leakage is the placement of the tracheal tube distal of the lesion in one mainstem bronchus. In our patient with tracheal rupture, the lesion was sealed by replacement of the tracheal cannula shortly above the carina. Positioning of bronchus-blockers proximal of the lesion (in absence of tracheal involvement), double-lumen tube placement, jet-ventilation and cardiopulmonary bypass may also be possible options [16–21]. Surgical repair includes pulmonary patch plasties and colopharyngoplasty that may provide promising results, although mortality remains high in this patient population [18–20].

Acute care diagnostic measures to estimate tissue lesions and edema includes EGD and CT providing strengths and weaknesses for each method. Before EGD and CT are being initiated, airway patency of the patient should be confirmed and monitored during the examination (pulseoximetry). EGD remains the diagnostic standard of injury classification after caustic ingestion [4, 7, 22]. It should be performed within 24 h after the injury and provides direct visualization of mucosal structures. However, it may not always be completed safely due to edema or incompliance (2 of our patients) and overestimates the extent of necrosis, which might lead to unnecessary surgery [23–25]. CT-based diagnostic may underestimate injury severity in the acute phase after ingestion but may support endoscopic findings and provides important information regarding pulmonary, mediastinal, splenic and pancreatic involvement [2, 26]. Moreover, CT may reveal considerable unexpected findings (2 of our patients). In the patients presented here, both, EGD and CT, was completed prior to the decision for emergency surgery.

Our data suggest that patients who underwent CT diagnostic were at high risk of being associated with fatal outcome. This might be explained by a higher likelihood of extended diagnostic in patients presenting with considerable injury severity, i.e. requiring tracheal intubation.

Indication for emergent surgical exploration usually depends on evidence of perforation and/or extension of necrosis. Clinical conditions i.e. metabolic acidosis, coagulation disorders and renal failure may also influence decision-making [27–29]. Surgical approach in our patients included laparotomy and consecutive (partial or total) gastrectomy (depending on the extent of injury), and thoracotomy followed by esophagectomy and cervical esophagostomy [30]. Further intestinal resection may be required in due course (late sequelae and stricture control). There might be cases when only supportive care is performed despite formal indication for surgery. We experienced three patients who underwent acute surgical exploration to control organ perforation whereas reconstructive surgery was avoided due to catastrophic conditions. Our data support the literature that emergency surgery and esophagectomy in particular is significantly associated with a bad prognosis after caustic ingestion [25].

We acknowledge the retrospective nature of this study and the low sample sizes of included patients as limitations. Furthermore, there was no standard protocol or treatment algorithm for specific diagnostic and therapeutic measures in our patients presented. In our patients, the need for tracheal intubation, CT-diagnostic, higher EGD score, tracheotomy, and surgical interventions were significantly associated with mortality. Due to small sample sizes and the retrospective study design, these parameters may be interpreted as markers of the severity of the underlying disease rather than real independent predictors of mortality. Prospective randomized studies in patients with caustic ingestion would be desirable but there are many confounders that may not be addressed appropriately in these settings because of low incidence, incalculable high variability of volumes, concentrations and natures of ingested agents, and predisposing morbidity.

## Conclusions

Caustic ingestion in adult patients requires an ED infrastructure providing 24/7-availability of expertise in establishing emergent airway safety, endoscopic examination (EGD and bronchoscopy), and CT-diagnostic, intensive care and emergency esophageal surgery. We recommend that even in patients with apparently stable clinical conditions, careful monitoring of respiratory functions should be considered as long as diagnostic work-up is completed.

## Abbreviations

CT: computed tomography
ED: Emergency Department
EGD: esophagogastroduodenoscopy
EMS: emergency medical service
GI: gastrointestinal
ICD 10: International Statistical Classification of Diseases and Related Health Problems, revision 10
ICU: intensive care unit
SD: standard deviation
